# Primary Care: Mental and Behavioral Health and Persons with Intellectual and Developmental Disabilities

**DOI:** 10.3389/fpubh.2014.00076

**Published:** 2014-07-07

**Authors:** David A. Ervin, Ashley Williams, Joav Merrick

**Affiliations:** ^1^The Resource Exchange, Colorado Springs, CO, USA; ^2^National Institute of Child Health and Human Development, Jerusalem, Israel; ^3^New Heights Behavioral Health, Colorado Springs, CO, USA; ^4^Department of Psychology, University of Colorado at Colorado Springs, Colorado Springs, CO, USA; ^5^Health Services, Division for Intellectual and Developmental Disabilities, Ministry of Social Affairs and Social Services, Jerusalem, Israel; ^6^Division of Pediatrics, Hadassah Hebrew University Medical Center, Jerusalem, Israel; ^7^Kentucky Children’s Hospital, University of Kentucky College of Medicine, Lexington, KY, USA; ^8^Center for Healthy Development, School of Public Health, Georgia State University, Atlanta, GA, USA

**Keywords:** public health, disability, intellectual disability, mental health, behavioral health, primary care, integration

## Abstract

**Introduction:** There are multiple ways to address the mental and behavioral health needs of people with intellectual and developmental disabilities (IDD).

**Method:** In this paper, we do not argue for a particular approach or set of approaches, but instead review the benefits of integrating mental and behavioral health supports with primary healthcare based primarily on our experience in and understanding of healthcare systems in the United States. It is estimated that between 35 and 40% of people with IDD also live with psychiatric disorders. NADD, an association for persons with developmental disabilities and mental health needs in the US holds that coexisting IDD and a psychiatric disorder interferes with a person’s education and job readiness, and disrupts family and peer relationships. Historically, the presence of such disorders among people with IDD was not well understood or was discounted altogether.

**Conclusion:** Over the past 15 years, however, greater attention is being paid to these comorbidities and their treatment, including the need to integrate mental and behavioral health treatments into primary care. Healthcare must account for multiple domains of quality of life, going beyond yearly physicals, and acute care visits, for example, to assess individuals’ healthcare goals and support them in achieving those goals. While integrated healthcare delivery systems can be difficult to find and access for people with IDD, such approaches are more responsive to the comprehensive needs and desires of people with IDD.

## Introduction

The notion of integration in the context of physical and mental health is not new. More than 100 years ago, pioneer psychologist Alfred Adler (1870–1937) codified concepts that would form the foundation of individual psychology (sometimes called Adlerian Psychology). This theoretical frame holds that a person is “indivisible,” and that to achieve what today would be referred to as quality of life, he or she must achieve harmony in her or his relationships across all aspects of life. Accordingly, humans are whole units versus collections of component parts. Overlaying this construct onto healthcare allows practitioners to examine health status as a whole. The relationship between physical health and mental health is, accordingly, inextricable.

The American Association on Intellectual and Developmental Disabilities (1) has declared that “all people, including people with intellectual and developmental disabilities (IDD), should have timely access to high quality, comprehensive, accessible, affordable, appropriate healthcare that meets their individual needs, maximizes health, well-being and function, and increases independence and community participation.” Healthcare practitioners and those interested in providing high-quality care to this population must consider how to go about achieving the best possible healthcare interventions that result in improvements in health and quality of life, defined as the optimization of several life domains, including rights, relationships, satisfaction (feelings of well-being), environment (safety and adaptations), economic security, social inclusion, individual control, privacy, health, and growth and development (2). Adequate health is critical to the capacity of people to pursue interests and activities that can enhance other quality of life indicators.

Research demonstrates the many real and potential benefits of integrated care (3, 4). There are different forms of integration, from situations in which practitioners work from different sites and specialties, rarely interact, and are primarily equipped to work with individuals with uncomplicated diagnoses, to those where care providers work in a coordinated fashion, utilizing the same framework of care, consistently interacting with one another and developing treatment plans that cover the full spectrum of bio/psycho/social needs of their patients. Individuals with IDD are uniquely positioned to benefit from the coordination of care that emanates from integrated medical and mental and behavioral healthcare, as well as other specialty care disciplines, long-term services and supports, and other health, health promotion and wellness support systems and providers.

A recent study found over 40% of a cohort of 1,318 adults with IDD were diagnosed with four or more comorbidities, including 18% diagnosed with anxiety disorder and 17.8% with depression (5). People with IDD typically live within a family, which may be biological, foster, or adopted, or are supported in group living arrangements. The individual is also embedded within a community service delivery system that includes social workers, educators, clinical professionals, day and residential program providers, to name a few, and is supported by an interdisciplinary team (IDT). If and when an IDT, which should certainly include the healthcare practitioners who are supporting the person, can coordinate its care, reductions in duplications of effort, miscommunications among IDT members, and more effective treatment across the person’s support system results. Across a host of clinical and programmatic disciplines that are likely to comprise the person’s array of supports and should be coordinated, medical and mental and behavioral health supports are critical.

The need for integrated primary and mental and behavioral health can also be seen in the rapidly evolving demographics of the community of people with IDD. According to 2014 Centers for Disease Control and Prevention data, about 1 in 68 children has been identified with autism spectrum disorder. In the US, this is a 30% increase from 2008 to 2010. We are also aware of an estimated 850,600 people with IDD age 60 and older living in the community (6, 7) in the US, and their numbers will likely double over the next two decades as members of the “baby boom” generation reach retirement age. This is an unprecedented development inasmuch as the average life expectancy of people with developmental disabilities was just 22 years in 1931, compared to 59 years in 1976, and 66 years in 1993. At present, the causes of death for all individuals with IDD mirror those of the general population (i.e., coronary heart disease, type 2 diabetes, respiratory illnesses, and cancer), except for those with Down syndrome, who typically die earlier due to dementia-related causes (over half of those with Down syndrome are expected to live into their 50s and roughly 13% will reach age 65). One study found the average age of death for persons with IDD is now 63.3 years for males and 69.9 years for females (8, 9). These and other demographic trends indicate that our healthcare delivery systems and approaches must meaningfully coordinate primary, mental, and behavioral and other specialty care in order to address a variety of complex healthcare needs and the health status and outcomes people with IDD seek.

A range of benefits of integrating primary healthcare with mental and behavioral healthcare exists, including reduced costs, increased identification of mental health issues, increased accessibility to mental health services, and improved patient outcomes (10–15). Barriers to integrating care include reimbursement systems that preclude payment for co-visitation, the absence of care coordinators in healthcare delivery environments who understand people with IDD and the relationship of their health status and their mental and behavioral health needs, and resistance to the use of commonly accessible medical records to name just three.

## Integration

Traditionally, healthcare for individuals with IDD has been parsed out to multiple providers and/or agencies along disparate funding lines. Health, mental health, and behavioral providers are often housed separately and regulated and funded by different governmental entities. Bringing together those disciplines who have traditionally served individuals with IDD, while ideal, poses challenges to the *status quo* and is made more difficult by regulations and systems of financing that are based on diagnoses over actual need for or perceived benefits of particular services and interventions.

Throughout the US, certain mental and behavioral health services tend to be available to people because they have a psychiatric or mental health diagnosis, while other services are available to people in explicit relationship to their IDD. This approach misses the potential to make treatment services available based on the particular symptoms, needs, and desires of a person as a primary driver of service availability and delivery. While diagnoses are important, their presence or absence should not be the most important factor in whether or not services are available. People who are diagnosed with both an IDD and a mental health concern are far-too-frequently caught between two systems.

*Fourteen year-old Jacob is living at home with his parents. Jacob is diagnosed with autism spectrum disorder with moderate intellectual disability, attention deficit hyperactivity disorder (ADHD), and obsessive compulsive disorder (OCD). He is served in a Home and Community Based Services (HCBS) Medicaid Waiver program for children with autism, and attends public school. Jacob’s behaviors include occasional physical aggression and self-injurious behavior, hoarding anything of paper (e.g., newspapers, magazines, notebook paper, etc.), and hyperactivity. He is routinely monitored by his pediatrician, who treats his ADHD with Concerta. What is less clear is through what system Jacob receives mental and behavioral health supports. The local mental health system provider treats the OCD, but believes the aggression and self-injurious behavior to be manifestations of Jacob’s autism and intellectual disability. And, the mental health provider can and does receive Medicaid payment for treatment of mental health, but cannot receive Medicaid reimbursement if treating anything related to the Autism or intellectual disability. At the same time, Jacob is supported with Applied Behavior Analysis for the physical aggression and self-injurious behaviors through the HCBS Medicaid waiver, with yet another provider reimbursed for these particular supports. The school that Jacob attends provides in-school treatment supports that are available under special education*.

Fortunately, Jacob’s mental health and behavioral support providers, along with his IDT, collaborate in the development of treatment plans; however, this fragmented system of supports and sources of financial support to pay for them is emblematic of the challenges people with IDD who have mental or behavioral health support needs face as a function of related but distinct systems that are designed around diagnoses instead of needs.

Barriers to integration like those that are impacting Jacob and his family are, unfortunately, the norm. In Jacob’s case, he and his family must interact with his pediatrician for primary care, including treatment of his ADHD, with his mental health counselor and psychiatrist who are treating his OCD, and his behavior support specialist who designs Jacob’s interventions for aggression and self-injurious behavior. These three systems are not co-located, do not share a common electronic health record (EHR) through which to communicate approaches to treatment and outcomes, and apart from crisis situations, do not tend to exchange information or collaborate in any meaningful way. Furthermore, the two systems to which Jacob and his family look for help use different languages that reflect differences in approach to treatment. For example, the mental health system, generally, takes an orientation to rehabilitation. In a rehabilitation model, some form of “cure” to a particular illness or concern is an overarching objective. To rehabilitate a person is to restore them to some baseline, to assist them in regaining lost skills. This implies an end point at which the person has regained lost function. Alternatively, the traditional developmental disability orientation is to habilitation. Habilitation seeks to maintain skills, or to teach new or more functional skills. These distinct underpinnings to each system guide approaches to treatment, are the basis of different financing systems, and create different treatment expectations. Most importantly, they create a distinct treatment framework that can be difficult for people with IDD and their families to navigate.

## Models of Integrated Care

Like Jacob, many individuals with IDD experience comorbidities of medical, mental/behavioral health, and psychosocial concerns (16). In 2009, Kronick et al. (17) found that 47% of Medicaid-only enrollees with a qualifying disability also were diagnosed with bipolar disorder, psychosis, depression, or another form of mental illness. In studying the most common clusters of health conditions among Medicaid-only enrollees with chronic disabilities, mental illness was included in three of the top five pairings among the highest-cost Medicaid beneficiaries (i.e., those in the upper 5% of the per capita cost range). For those with the most common chronic physical health conditions, healthcare spending is 60–75% higher for those with a mental illness than for those without one. These data combine to compel the development of models that integrate care and focus on the intersection of mental and behavioral health with primary care.

Developmental Disabilities Health Center (DDHC) in Colorado is health home to nearly 450 children and adults with IDD. Offering multidisciplinary care that features integrated primary and behavioral healthcare, the DDHC braids traditional primary care with behavioral health, mental health, and psychiatry. Through this model, patients see both medical and behavioral providers at their initial appointment to assess the physical as well as psychosocial needs of the patients. At subsequent appointments, behavioral health providers are called into medical appointments as necessary, and the development of a patient’s treatment plan reflects his or her general and behavioral health needs alike. Patients also have the option of scheduling follow-up appointments with behavioral health providers, who are on-site and can also support the individual in his or her community outside of the DDHC, or the psychiatrist. Behavioral health providers, which include a psychologist and three master’s level clinicians, offer a range of experiential therapies, dance/movement therapy, and behavioral therapy interventions, among others. Patients of DDHC are referred for these services when a model of brief episodic-driven therapy is not likely to address more long-standing mental health concerns. Clinic providers most often communicate live and in real time, but also use an EHR system that provides them a means for communicating and coordinating care on a common platform.

Another very promising integrated model is the DD Health Home (18) model, which offers specialized treatment designed for people with IDD. This model integrates mental health services, including diagnostic assessment and treatment, with a range of services typically associated with primary care. People with IDD who are diagnosed with psychiatric conditions “receive coordinated care, monitoring for medication interactions and side effects, as well as regular review of the effectiveness of all relevant treatments as part of their routine care” (pp. 16–17). The efficacy of the model, as measured by a range of factors – from patient satisfaction to emergency room visits and hospitalizations – is compelling.

## Challenges to Care Integration

While the DDHC, the DD Health Home and other models have succeeded in integrating care and offering patient-centric, responsive, and holistic treatment, there are a number of barriers to access. As with our example of Jacob, sources of and rules that govern certain financing systems create silos in the delivery of care. In addition, practical limitations on integration of behavioral and mental health with primary care include:

- State Medicaid limitations on payments for same-day billing for a physical health and a mental health service/visit;
- Lack of reimbursement for collaborative care and case management related to mental health services;
- Absence of reimbursement for services provided by non-physicians, alternative practitioners, and contract practitioners and providers;
- Medicaid disallowance of reimbursement when primary care practitioners submit bills listing only a mental health diagnosis and corresponding treatment;
- Level of reimbursement rates in rural and urban settings;
- Difficulties in getting reimbursement for mental health services in-school-based health center settings; and,
- Lack of reimbursement incentives for screening and providing preventive mental health services in primary care settings (19).

In addition, different long-term services and support systems – for example, whether or not the person with IDD is supported by a residential provider or a family caregiver – may bring additional challenges to fuller integration. Raising awareness of a person’s IDT that healthcare providers seek and are invested in the well-being of patients sometimes can be an attitudinal barrier. A serious and additional barrier is lack of follow through with recommendations made by the medical and mental/behavioral health providers. Patients may leave an integrated healthcare encounter with referrals for specialty care, directions regarding increasing exercise and healthy eating habits, as well as goals to enhance social participation activities. It is rare that a provider will be able to achieve the full list of follow-up recommendations. This is particularly the case when the person with IDD is served by a residential provider, and frequently may be the result of lack of transportation, staff turnover, and inconsistencies in staff support, and other competing priorities that must also be addressed by the supporting staff person. Coordination of care among multiple responsible parties (e.g., host home provider, day program agencies, guardians, and service coordinators) is often challenging to any extent that it is unclear who is responsible for the steps in follow through for the various treatment objectives. This is made more challenging by varied and frequently conflicting sets of IDD and mental health system regulations. Furthermore, with multiple involved parties, a break in the chain of communication among any members of the IDT is likely to impede following through with these objectives.

A third limitation of collaborative and integrated care is cultural, and involves preexisting conceptions of what healthcare is designed to do for individuals with IDD, which fall short of current best practices in integrated healthcare. Primary care practitioners must accept that mental and behavioral health is a part of total health, that additional professionals such as psychiatrists, psychologists, and care coordinators are needed and valuable in primary care to change mental health outcomes for people, and that adjustments in work processes for both physical and mental health providers are required to address a person’s mental and behavioral health needs in what is otherwise a primary care setting (20). People with IDD, their caregivers and the people who support them, as well as physicians and other healthcare providers must consider proactive strategies for the improvement of health as the norm, versus procedures-focused, standard 15-minute encounters, automatic prescription of medications, particularly to address behavioral concerns, and benign attitudes when it comes to all different aspects of the development of quality of life for their patients.

## Conclusion

The challenges to integration of primary care with mental and behavioral health range from systemic to cultural. Overcoming these barriers requires work in public policy (e.g., addressing diagnoses-based eligibility standards that differ across mental health and developmental disability systems), financing and reimbursement systems (e.g., in the US, Medicaid state plan versus Medicaid waiver systems), and establishing new healthcare cultural norms through training of healthcare providers on the benefits of integrated care. However, the benefits of integrated care make for compelling reasons to address these challenges.

We know of the interaction of body and mind for people in general, let alone among people with complex comorbidities (21). We are also aware of the benefits of care integration found in the delivery of multidisciplinary, integrated healthcare to individuals with IDD. Practitioners and others interested in enhancing the well-being and health outcomes of individuals with IDD must continue to seek collaboration, not only among themselves, but across disciplines, to include all members of IDTs. To those ends, we offer the following recommendations to address obstacles to integrated care and to enhance and expand systems of care that offer integration and the benefits that accompany it:

- Merging the two distinctly different cultures that characterize the mental health and primary care systems and their practitioners can be a profound challenge and can impede successful integration of the two disciplines (22). To address needed cultural change in support of fuller integration of care, healthcare provider education and training programs must include training of practitioners on the diverse, often-complex, and unique care needs of individuals with IDD, while demonstrating the benefits of patient-centric integration of mental and behavioral healthcare with primary care. New evidence-based standards of integrated treatment, borne out of broad guidelines of care that are dedicated to children and adults with IDD, should be created and taught in medical schools and doctoral programs that are preparing next generation practitioners.
- Current predominant third party private and public reimbursement schemes discourage integration. Reimbursement for behavioral health, for example, has been fragmented, and regulations in many situations preclude billing for a behavioral health encounter on the same day as a primary care encounter. We recommend that payment models be reexamined to create incentives for collaborative care and outcomes. For example, global payment systems, which are non-encounter-based reimbursements that encourage a multidisciplinary approach to self-directed holistic care – where a person’s mental health needs can be treated and financially supported in the same payment as his or her primary care needs – have the potential to shift our orientation to a set of overall health outcome objectives.
- The use of EHR systems is increasing, particularly since January 2009 at the passage in the United States of the Health Information Technology for Economic and Clinical Health (HITECH) Act. These systems afford an unlimited number of healthcare providers access to up-to-date patient information in real time; and, these EHR systems offer those same providers the means to communicate and coordinate their treatment. However, EHR systems are limited. If two separate practices, each with a healthcare provider that serves the same patient, use two different systems, providers cannot communicate and coordinate their care by those means. It is recommended that EHR platforms be enhanced to allow for multiple system integration. For example, if a primary care clinic uses NextGen and the behavioral health provider uses CarePaths, these two systems should integrate to create a consolidated record for common patients that is accessible to their treating providers. In addition, it is recommended that governments invest in and create incentives for the private sector’s adoption and implementation of EHR systems as a priority in public policy. The HITECH Act of 2009 in the US appropriated over $19 billion to increase the use of EHR systems, and may be a model for other countries.
- Regulations that are built around particular diagnoses, which in turn define what systems can deliver particular services, need to be rethought and modernized. With as many as 40% of people with IDD also living with a mental health concern, regulations need to allow for seamless movement within and between systems – without creating barriers to reimbursements for treating providers. Further, regulations that have historically created siloes across the IDD, mental health, school, healthcare, and other systems need to reflect a priority for self-direction and self-determination, and enable people with IDD and their families the latitude to make decisions about how best to access and in what system they seek care.

People with IDD, their families and advocates, and a growing number of healthcare providers are pushing for the development of more and better models of integrated care. We are under no illusion that needed changes to the way in which we think about and deliver primary care and mental and behavioral health will be easy. Systems of care and the cultures that characterize them are deeply entrenched. However, interest in healthcare integration has grown in recent years with a rapid escalation in healthcare costs and mounting evidence of the role of integration in slowing cost increases and better outcomes for people. In the US, the Patient Protection and Affordable Care Act (PPACA) encourages clinical integration through its support of innovative models of care that promote partnership among healthcare providers. The PPACA also has paved the way for states to establish Health Homes to coordinate care for people with Medicaid who have chronic conditions. Designed to address the whole person, this Health Home model will integrate and coordinate all primary, acute, behavioral health, and long-term services and supports. Models that are customized to the needs of people with IDD, such as New Jersey’s DD Health Home and Colorado’s DDHC, are yielding promising outcomes and, critically, are replicable. Continued expansion of similar and the development of new systems that build on these and other models can improve availability and access, and, most importantly, result in greatly improved quality of life for people with IDD.

## Conflict of Interest Statement

The authors declare that the research was conducted in the absence of any commercial or financial relationships that could be construed as a potential conflict of interest.

## References

[1] American Association on Intellectual and Developmental Disabilities. Declaration on health parity for persons with intellectual and developmental disabilities. Joint Position Statement on Health, Mental Health, Vision, and Dental Care. Washington, DC Available from: http://aaidd.org/news-policy/policy/position-statements/health-mental-health-vision-and-dental-care#.UnKl5rLnZdg

[2] Keith KD Heal LW Schalock RL Cross-cultural measurement of critical quality of life concepts. J Intellect Dev Disabil (1996) 21(4):273–93 10.1080/13668259600033201

[3] Goddell S Druss B Reisinger WE Mental Disorders and Medical Comorbidity. Princeton, NJ: Robert Wood Johnson Policy Brief (2011).

[4] Jansen DEMC Krol B Groothoff JW Post D Towards improving medical care for people with intellectual disability living in the community: possibilities of integrated care. J Appl Res Intellect Disabil (2006) 19:214–8 10.1111/j.1468-3148.2005.00255.x

[5] Rimmer JH Hsieh K Longitudinal Health and Intellectual Disability Study (LHIDS) on obesity and health risk behaviors. Proceedings of the Lifespan Health and Function of Adults with Intellectual Disabilities: Translating Research into Practice, State of the Science Conference, Bethesda, MD (2011)

[6] Larson SA Lakin KC Anderson L Nohoon K Lee JH Anderson D Prevalence of mental retardation and developmental disabilities: estimates from the 1994/1995 National Health Interview Survey Disability Supplements. Am J Ment Retard (2001) 06(3):231–52 10.1352/0895-8017(2001)106<0231:pomrad>2.0.co;2 11408960

[7] US Census Bureau. DP-1 – United States: Profile of General Population and Housing Characteristics: 2010, 2010 Demographic Profile Data. Available from: http://www.aoa.gov/AoARoot/Aging_Statistics/Census_Population/census2010/Index.aspx

[8] Walker L Rinck C Horn V McVeigh T Aging with Developmental Disabilities: Trends and Best Practices. Kansas City, MO: University of Missouri Kansas City (2007).

[9] Long T Kavarian S Aging with developmental disabilities: an overview. Top Geriatr Rehabil (2008) 24(1):2–11 10.1097/01.TGR.0000311402.16802.b1

[10] Funk M Saraceno B Drew N Faydi E Integrating mental health into primary healthcare. Ment Health Fam Med (2008) 5(1):5–8 22477840 PMC2777555

[11] Katon WJ Roy-Byrne P Russo J Cowley D Cost-effectiveness and cost offset of a collaborative care intervention for primary care patients with panic disorder. Arch Gen Psychiatry (2002) 59(12):1098–104 10.1001/archpsyc.59.12.1098 12470125

[12] Kates N McPherson-Doe C George L Integrating mental health services within primary care settings: the Hamilton Family Health Team. J Ambul Care Manage (2011) 34(2):174–82 10.1097/JAC.0b013e31820f6435 21415615

[13] Bryan CJ Morrow C Appolonio KK Impact of behavioral health consultant interventions on patient symptoms and functioning in an integrated family medicine clinic. J Clin Psychol (2009) 65(3):281–93 10.1002/jclp.20539 19152340

[14] Katon W Von Korf fM Lin E Simon G Walker E Unützer J Stepped collaborative care for primary care patients with persistent symptoms of depression: a randomized trial. Arch Gen Psychiatry (1999) 56(12):1109–15 10.1001/archpsyc.56.12.1109 10591288

[15] Katon WJ Von Korff M Lin EH Simon G Ludman E Russo J The Pathways Study: a randomized trial of collaborative care in patients with diabetes and depression. Arch Gen Psychiatry (2004) 61(10):1042–9 10.1001/archpsyc.61.10.1042 15466678

[16] Naaldenberg J Kuijken N van Dooren K van Schrojenstein H de Valk L Topics, methods and challenges in health promotion for people with intellectual disabilities: a structured review of literature. Res Dev Disabil (2013) 34(12):4534–45 10.1016/j.ridd.2013.09.029 24161461

[17] Kronick RG Bella M Gilmore TP The Faces of Medicaid III: Refining the Portrait of People with Multiple Chronic Conditions. Trenton, NJ: Center for Health Care Strategies (2009).

[18] Kastner TA Walsh KK Health care for individuals with intellectual and developmental disabilities. Int Rev Res Dev Disabil (2012) 43:1–45 10.1016/B978-0-12-398261-2.00001-5

[19] Kautz C Mauch D Smith SA Reimbursement of Mental Health Services in Primary Care Settings. Rockville, MD: Center for Mental Health Services (2008).

[20] Kathol RG Butler M McAlpine DD Kane RL Barriers to physical and mental condition integrated service delivery. Psychosom Med (2010) 72(6):511–8 10.1097/PSY.0b013e3181e2c4a0 20498293

[21] Smith GC From consultation-liaison psychiatry to integrated care for multiple and complex needs. Aust N Z J Psychiatry (2009) 43(1):1–12 10.1080/00048670802534358 19085523

[22] Institute for Health Care Improvement. IHI 90-Day R&D Project Final Summary Report: Integrating Behavioral Health and Primary Care. Cambridge, MA: Institute for Health Care Improvement Available from: http://www.ihi.org/resources/Pages/Publications/BehavioralHealthIntegrationIHI90DayRDProject.aspx

